# Inhibition of insulin-regulated aminopeptidase confers neuroprotection in a conscious model of ischemic stroke

**DOI:** 10.1038/s41598-023-46072-5

**Published:** 2023-11-13

**Authors:** Jonathon Telianidis, Andrew Hunter, Robert Widdop, Barbara Kemp-Harper, Vi Pham, Claudia McCarthy, Siew Yeen Chai

**Affiliations:** 1 Department of Physiology, Monash Biomedicine Discovery Institute, Monash University, Clayton, VIC 3800 Australia; 2 Department Pharmacology, Monash Biomedicine Discovery Institute, Monash University, Clayton, VIC 3800 Australia; 3 Drug Discovery Biology, Monash Institute of Pharmaceutical Sciences, Monash University, Parkville, VIC 3052 Australia

**Keywords:** Neuroscience, Neurology

## Abstract

Stroke is a leading cause of mortality and morbidity with a paucity of effective pharmacological treatments. We have previously identified insulin-regulated aminopeptidase (IRAP) as a potential target for the development of a new class of drugs for the treatment of stroke, as global deletion of this gene in mice significantly protected against ischemic damage. In the current study, we demonstrate that small molecular weight IRAP inhibitors reduce infarct volume and improve neurological outcome in a hypertensive animal model of ischemic stroke. The effects of two structurally distinct IRAP inhibitors (HFI419 or SJM164) were investigated in a model of stroke where the middle cerebral artery was transiently occluded with endothelin-1 in the conscious spontaneously hypertensive rat. IRAP inhibitor was administered into the lateral ventricle at 2 or 6 h after stroke, with subsequent doses delivered at 24, 48 and 70 h post-stroke. Functional outcomes were assessed prior to drug treatment, and on day 1 and 3 post-stroke. Histological analyses and neuroinflammatory cytokine profiling were conducted at 72 and 24 h post-stroke respectively. IRAP inhibitor treatment following stroke significantly reduced infarct volume and improved neurological and motor deficits. These protective effects were maintained even when the therapeutic window was extended to 6 h. Examination of the cellular architecture at 72 h post-stroke demonstrated that IRAP expression was upregulated in CD11b positive cells and activated astrocytes. Furthermore, IRAP inhibitor treatment significantly increased gene expression for interleukin 6 and C–C motif chemokine ligand 2 in the ischemic core. This study provides proof-of-principle that selective inhibition of IRAP activity with two structurally distinct IRAP inhibitors reduces infarct volume and improves functional outcome even when the first dose is administered 6 h post-stroke. This is the first direct evidence that IRAP inhibitors are a class of drug with potential use in the treatment of ischemic stroke.

## Introduction

Ischemic stroke constitutes approximately 80% of all cerebrovascular events and is one of the leading causes of death and disability worldwide^1^. The only effective pharmacological therapeutic intervention is the lysis of the clot with intravenous administration of recombinant tissue plasminogen activator (tPA). However, this is only effective when given within 4–5 h of the ischemic event^2,3^ or up to 9 h with favorable computed tomography perfusion imaging^4^. More recently, endovascular thrombectomy with concomitant intravenous or intra-arterial tPA has proven efficacious in improving stroke outcome and reducing disability^5–9^. Despite the efficacy of current stroke therapies, accessibility and administration is time constrained, subject to a strict inclusion criterion and require specialized centers. Furthermore, the therapeutic mechanism is limited to restoring central perfusion which is not without associated risks, including reperfusion injury and hemorrhage. As such, adjunct neuroprotective therapies offer a potentially critical role in acute stroke care firstly by way of preserving the ischemic penumbra and extending the therapeutic window of the current therapies. Secondly, as a standalone therapy, a pharmacological intervention that could arrest the deleterious processes of the ischemic and inflammatory cascade, as well as promoting neuronal survival and repair would benefit patients who would otherwise be excluded from current best therapies^10^. With this in mind, we have recently identified a role for insulin-regulated aminopeptidase (IRAP) in the pathogenesis of focal ischemic damage^11^.

IRAP is a type II integral membrane protein that belongs to the M1 aminopeptidases that was first identified in muscle and adipose tissue and has strong associations with the insulin-sensitive glucose transporter GLUT4 within specialized vesicles^12^. IRAP is expressed in a number of tissue types and found in specific neuronal populations^13^ as well as various immune cells including monocytes^14^, mast cells^15^, and dendritic cells^16^. IRAP contains two functional domains. The N-terminal cytoplasmic domain is a critical regulator of vesicular trafficking and sorting of GLUT4 from endosomes to GLUT4-specialised vesicles^17,18^. The much larger C-terminal catalytic domain contains the substrate recognition motif important for the selective processing of neuropeptides including oxytocin, vasopressin, somatostatin, lys-bradykinin (kallidin) and met-enkephalin as well as a zinc binding motif characteristic of many metallopeptidases^19,20^. Naturally occurring peptide inhibitors of this enzyme such as angiotensin (Ang) IV and LVV-hemorphin 7 enhance learning and memory in the healthy rodent^21–23^ as well as in several animal models of cognitive impairment^24–27^. More recently, the effect of enhanced learning and memory elicited, were replicated using the specific small molecular weight non-peptide IRAP inhibitor HFI419^28,29^.

In the phenotypic characterization of global IRAP knockout (KO) mice, we observed that these mice were protected against focal ischemic damage^11^, as well as epileptiform activity^30^, a common post-stroke complication^31^. In the IRAP KO mice, ischemic damage resulting from transient occlusion of the middle cerebral artery (MCA) was 80% less compared to wild type animals. Furthermore, this translated into a concomitant improvement in neurological performance^11^.

The current study aims to obtain proof-of-principle that the inhibition of IRAP activity with either of the two structurally distinct, small molecular weight and specific inhibitors of the enzyme, HFI149 and SJM164, protects against focal ischemic damage in a conscious model of stroke using the spontaneously hypertensive rat (SHR). We provide compelling histological and functional evidence on the benefit of IRAP inhibition following cerebral ischemia and therefore highlight IRAP as a promising new target for the treatment of stroke.

## Methods

### Ethics statement

All animal procedures were performed in accordance with the National Health and Medical Research Council of Australia guidelines with approval from Monash University Animal Ethics Committee. No experiments on humans or human tissue was conducted.

### Animals

As this is a proof of principle study, we elected to use only male rats. Male spontaneously hypertensive rats (SHR) (N = 130) purchased from Animal Resource Centre (Western Australia) were 20–24 weeks (350–400 g) at the time of experimentation. The rats were housed individually with access to standard rat chow (Barastoc Ridely, Australia) and water ad libitum. The housing facility followed a 12 h day/night cycle. The study was completed in accordance with ARRIVE guidelines^32^. At the end of the experimental timeline (Supplementary Fig. 1A), animals were anesthetized with ketamine (80 mg/kg) and xylazine (10 mg/kg) and decapitated while heavily anaesthetized.

### Surgical procedures

The SHRs were anaesthetized with ketamine (75 mg/kg; Sigma)/xylazine (10 mg/kg; Troy; i.p) for the stereotaxic implantation of a 23-gauge stainless steel guide cannula into the right piriform cortex (coordinates: 0.2 mm anterior, − 4.7 mm lateral relative to bregma, and – 7 mm ventral relative to the skull surface) to sit 3 mm dorsal to the right middle cerebral artery^33–35^. An additional 23-gauge guide cannula was implanted into the left lateral ventricle (− 0.8 mm anterior, + 1.5 mm lateral relative to bregma and − 3.2 mm ventral relative to the skull surface) for the intracerebroventricular administration of IRAP inhibitor or vehicle control. This was followed by a 5-day recovery period prior to stroke induction.

### Stroke induction

Stroke was induced in the conscious SHR by titration of the potent vasoconstrictor endothelin-1 (ET-1) (2 pmol/µl in saline; AusPep) at a rate of 0.2 µl every 30 s, up to 7 µl, through a 30-gauge injector protruding 3 mm beyond the end of the previously implanted guide cannula. Stroke severity was characterized by behavioral indicators described below and ET-1 was infused until a desired level of stroke was achieved. The stroke severity was graded on a pre-determined scale of 1–4. Level 1 graded stroke represents a mild stroke while a level 4 graded stroke represents a severe stroke, based on previously published behavioral criteria^34,36^. Typical changes in behavior include grooming, teeth chatter, contralateral and ipsilateral spinning, clenching or dragging of contralateral forepaw, and jaw flexion or biting with the latter 2 indicative of a grade 4 stroke. Only animals with a stroke grading of level 4 were used for this study (N = 87). Reperfusion begins 20 min following cessation of ET-1 delivery with cerebral blood flow reaching 90% at approximately 60 min followed by a gradual reperfusion lasting 16–22 h^37,38^. Rectal temperature and observations of habitual behaviours were monitored every 30 min for 3 h following stroke. No animals exceeded a stroke grade greater than level 4.

### Drug preparation and treatments

All drugs were prepared in a vehicle solution comprising of 2.5% dimethyl sulfoxide (DMSO) in saline. The SHR that underwent stroke induction were randomly allocated to the following treatment groups: vehicle, 0.1 or 1 nmol HFI419 or 1 nmol SJM164. These drug doses were selected as they have been previously shown to enhance memory with intracerebroventricular administration^28^. A 2 µl bolus of either IRAP inhibitor (HFI419 or SJM164) or vehicle was administered into the left lateral ventricle at the following time points: 2 or 6, 24, 48 and 70 h post-stroke. Drug vials were labeled with non-identifying terms by a separate researcher prior to administration. Sham animals underwent cannula implantation surgery and were administered saline during stroke induction and vehicle solution at the treating timepoints.

### Neurological deficit assessment

Prior to stroke, and at 24 and 70 h post stroke, neurological deficit was assessed. Stroke-induced hemiplegia was evaluated by gently suspending the rat from the tail and measuring postural and thoracic flexion and rotation as well as the degree of contralateral forelimb extension. This technique has previously been described^39–41^. A score between 0 to 3 was assigned to grade each deficit category (thoracic flexion/rotation and contralateral paw extension), with the sum of both scores used to give a measure of the severity of neurological deficit. A higher score corresponds to a greater neurological deficit whereas a score of zero indicated no detected neurological deficit. Body weight was monitored pre-stroke as well as 24 h and 70 h post-stroke to provide a general measure of health.

### Ledge-beam motor function assessment

Changes in motor function was examined using the ledge beam test where the animals were required to traverse a gradually narrowing ledged beam inclined at an angle of 30°. Stroke-induced motor deficits were detected as rear foot faults onto the under-hanging ledge of the beam and quantified as a percentage of the total number of steps taken to cross the beam or % error^34^. SHR were trained on two separate occasions (-4 and -3 days) prior to surgery to traverse the beam without stepping onto the under-hanging ledge. Prior to stroke induction, and at 24 and 70 h post-stroke, SHR were tested in three consecutive trials and the number of contralateral rear foot faults and total steps taken were determined. The data is expressed as the mean percentage of foot faults for the trial period.

### Rotarod assessment

On day 3 post stroke, SHR were placed on the rotarod consisting of a motorized cylindrical assembly of 18 stainless steel rods capable of rotating at a maximum speed of 30 revolutions per minute (30 rpm; Ratek, Australia). Top speed of three attempts was used to determine a final score, to maintain an upright walking position and qualify as a successful completion, an animal had to grip the rotating rods at each speed for 3 s. The rotating speed began at 6 rpm and was raised 1.5 rpm every 3 s until the animal lost an upright walking position or until the maximum 30 rpm was reached.

### Systolic blood pressure measurement

A small subset from each treatment group (n = 3–5) were used to determine if stroke or drug treatment influenced blood pressure regulation. Systolic blood pressure was measured via a non-invasive, tail-cuff pressure analysis system (Model MC400, Hatteras Instruments) where the SHR were placed in a restrainer on a heated platform. The SHR were habituated to the restrainer 1 day before the first systolic blood pressure measurement. Systolic blood pressure was measured 5 days prior to stroke and 70 h post-stroke with the procedure taking 5–10 min to complete. Each single measure represents an average of 5 systolic measurements.

### Infarct and forebrain volume quantification

At 72 h after the induction of cerebral ischemia, SHR were anesthetized with ketamine (75 mg/kg; Sigma)/xylazine (10 mg/kg; Troy; i.p) and transcardially perfused with phosphate buffered saline (10 mM sodium phosphate and 150 mM sodium chloride; pH 7.4). The brains were removed, snap frozen in liquid nitrogen and sectioned in a cryostat at 16 µm thickness in the coronal plane at the following 8 predetermined levels: + 2.10 mm, + 1.18 mm, + 0.74 mm, − 0.10 mm, − 1.06, − 1.82 mm, − 2.70 mm and − 3.88 mm relative to bregma. The sections were then imaged and the infarct volume was calculated using the ballistic light method^42^. Using ImageJ, the total infarct volume was quantified by integrating the area of infarct at each level with the distance between each level^43^. Correction for oedema was calculated as previously described^42^. The same sections were used to determine hemispheric and total brain volumes as a marker of forebrain oedema. The hemispheric volumes were calculated as a percentage change (PC) in which the ipsilateral hemisphere (IH) was compared to the contralateral hemisphere (CH) using the formula: PC = ((IH/CH)*100) − 100.

### Immunohistochemistry

Coronal sections taken at + 0.74 mm relative to bregma were post-fixed for 15 min in 4% paraformaldehyde followed by three washes in phosphate buffered saline (PBS) for 10 min each. The sections were then incubated in blocking solution (0.3% Triton-X-100 and 10% normal donkey serum (NDS) prepared in PBS) for 1 h at room temperature. This was followed by an overnight incubation with one of the following antibodies: NeuN for neurons (1:500 dilution, Invitrogen), glial fibrillary acidic protein (GFAP) for activated astrocytes (1:500 dilution, Invitrogen), CD11b (1:500 dilution, Serotec), GLUT-1 for cerebral microvessels (1:500 dilution, Millipore) or IRAP (1:500 dilution, Invitrogen) at 4 °C. All primary antibodies were diluted in 0.3% (v/v) Triton-X-100 and 3% (v/v) NDS prepared in PBS. Slides were washed three times with 0.1% (v/v) Tween-20 prepared in PBS before being incubated with an Alexa-Fluor® fluorescently conjugated secondary antibody 1:500 (Alexa-Fluor® 488 or 568) for 1 h at room temperature. All secondary antibodies were diluted in 0.3% (v/v) Triton X-100 and 3% (v/v) NDS prepared in PBS. Slides were washed three times with 0.1% (v/v) Tween-20 prepared in PBS and then coverslipped with DAKO Fluorescence Mounting Medium. The regions imaged include the cortical and striatal ischemic core, representing infarcted tissue, and the cortical penumbra which represents mildly damaged but salvageable tissue surrounding the ischemic core (Supplementary Fig. 1B). In addition, the respective matched contralateral regions were imaged. For each brain section, three images were captured within each brain region. Single images for animals treated with an IRAP inhibitor beginning at 2 h post stroke was captured using a Zeiss LSM700 inverted confocal microscope (Carl Zeiss Microscopy GmbH, Jena, Germany). A 0.56 mm^2^ field of view was used and NeuN, GFAP and CD11b immuno-positive cells were counted using Image J software and cell counts were averaged over the 3 images per brain region for each animal. For animals treated with an IRAP inhibitor beginning at 6 h post stroke, images were captured using the Leica® Aperio Scanscope AT Turbo slide scanner where a 1 mm^2^ field of view was used to count NeuN, GFAP and CD11b immuno-positive cells.

### Real-time PCR (RT-qPCR)

As treatment effects were evident as early as 24 h post stroke, in a subgroup of SHRs, we examined 24 h mRNA expression of inflammatory chemokines and cytokines with RT-qPCR. Rats treated with either vehicle or 0.1 nmol HFI419 2 h post-stroke, were anesthetized with ketamine (75 mg/kg; Sigma)/xylazine (10 mg/kg; Troy; i.p), decapitated and the whole brain removed. The brain was dissected into striatal core (whole striatum), cortical core (ventral cortex) and cortical penumbra (dorsal cortex) as well as the respective contralateral unlesioned matched regions. 25 mg of RNA was extracted from each dissected brain region and stored at – 80 °C using Trizol reagent (Life Technologies) according to the manufacturer’s protocol. The quantity and quality of the RNAs were determined using A260/A280 readings by NanoPhotometer (Implen). For RT-PCR, 2 mg of RNA was reverse transcribed into cDNA using High Capacity cDNA Reverse Transcription Kit (Applied Biosystems). The synthesized cDNA was used as a template for PCR reactions using TaqMan Fast Advanced Master Mix (Applied Biosystems). The TaqMan probes have the following identification number: 45S (Rn03928990_gl), GFAP (Rn00566603_m1), CD11b (Rn00709342_m1), TNF-⍺ (Rn00562055_m1), IL6 (Rn01410330_m1), BDNF (Rn01484924_m1), IL-1b (Rn00580432_m1), CCL2 (Rn00580555_m1), iNOS (Rn00561646_m1), TGF-β (Rn00572010_m1) and eNOS (Rn02132634_s1). Relative quantification of gene expression was performed by comparative threshold (CT) calculated by the 2^−ΔΔCT^ method^44^. Changes in mRNA expression levels were calculated following normalization to the house-keeping gene 45S.

### Wire myograph

Wire myograph was used to investigate blood vessel contractility in response to increasing doses of IRAP inhibitor. A subgroup of naïve SHR (n = 5) were anesthetized with ketamine (75 mg/kg; Sigma)/xylazine (10 mg/kg; Troy; i.p), decapitated and the whole brain removed. Fresh basilar arteries were dissected and placed in a cold carbogen-bubbled (95% O_2_, 5% CO_2_) solution of Krebs-bicarbonate (KB; composition in mM NaCl 118, KCl 4.5, MgSO_4_ 0.45, KH_2_PO_4_ 1.03, NaHCO_3_ 25, glucose 11.1, CaCl_2_ 2.5). Arteries were cut into 2 mm segments and mounted in a Mulvany-Halpern myograph (Danish Myo Technology A/S) via two 40 µm wires. Arteries were equilibrated at 37 °C for 30 min, washed with KB and then allowed to rest for 10 min before tensioning to 5 mN. Arteries were maximally contracted with high potassium salt solution (KPPS; composition containing 122.5 mM KCl as an equimolar replacement of NaCl with KCl), washed out with KB and then 30 min later sub-maximally contracted to 50% of maximal KPPS contraction with the thromboxane A2 receptor agonist U46619 (1 × 10^–9^–1 × 10^–7^). To ensure the viability of the endothelium, 10 µM of acetylcholine was added to assess relaxation. Preparations with a relaxation percentage of 65 or higher of maximal contraction were used. Once the contractions were stable, either HFI419 (1 × 10^–12 ^– 1 × 10^−6^M) or vehicle (time control) was delivered in cumulative concentrations. To ensure maximal relaxation could be achieved, sodium nitroprusside (SNP; 1 × 10^–5^ M) was added to each preparation at the conclusion of the experiment.

### Statistical analysis

Data are expressed as the mean ± SEM unless stated otherwise with statistical analysis completed using GraphPad Prism 9. Infarct volume was analysed with a one-way ANOVA followed by a Dunnett’s test for drug treatment beginning at 2 h post stroke or with an unpaired t-test for drug treatment beginning at 6 h post stroke. The neurological score, ledge-beam test, immunohistochemical cell counts, RT-qPCR, hemispheric brain volume and wire myograph were analysed with a two-way repeated measures ANOVA followed by a Tukey’s test. The sample size and *P* values are provided with each figure with a *P* < 0.05 considered significant.

## Results

### IRAP inhibitor treatment with HFI419 or SJM164 significantly reduced infarct volume even when the first dose was delayed to 6 h post-stroke

**Figure 1:**
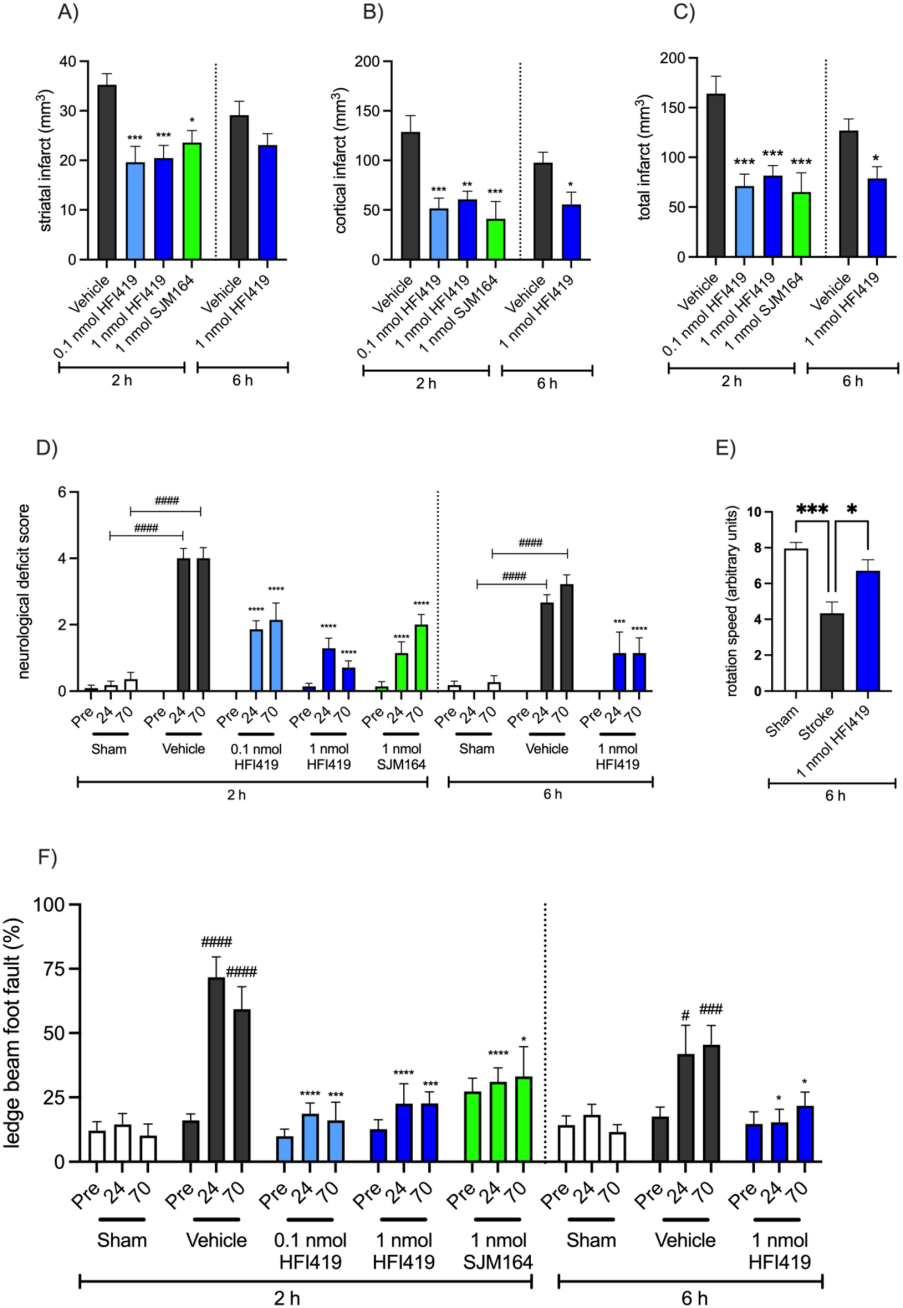
Inhibition of IRAP reduced infarct volume and improved post-stroke motor and neurological function. Treatment with either 0.1 or 1 nmol HFI419 or, 1 nmol SJM164 at 2, 24, 48 and 70 h post-stroke significantly reduced (**A**) striatal, (**B**) cortical and (**C**) total infarct. Delaying first treatment of 1 nmol HFI419 to 6 h post-stroke, followed by treatment at 24, 48 and 70 h post-stroke reduced (**B**) cortical and total (**C**) infarct volume (n = 7–10, *P < 0.05, **P < 0.01, ***P < 0.001 vs. vehicle; the data represented as mean ± SEM and analysed by one-way ANOVA followed by a Dunnett’s test or unpaired t-test). (**D**) Neurological deficits were significantly improved following treatment with an IRAP inhibitor beginning at 2 or 6 h post-stroke, where a higher score indicates greater neurological deficit (pre = pre-stroke, 24 = 24 h post-stroke, 70 = 70 h post-stroke; n = 7–14, #*P* < 0.05, ##*P* < 0.01, *P* < 0.0001 vs time-matched sham, *P < 0.05, ***P < 0.001, ****P < 0.0001 vs. time-matched vehicle; data was analysed with a two-way repeated-measures ANOVA followed by a Tukey’s test; n = 5–11). Motor function was significantly improved following treatment with an IRAP inhibitor. (**E**) Treatment with 1 nmol HFI419 beginning at 6, 24, 48 and 70 h post-stroke significantly improved performance on the rotarod compared to vehicle treatment (n = 6–10, *P < 0.05, ***P < 0.001, analysed by one-way ANOVA followed by a Dunnett’s test). (**F**) IRAP inhibition beginning at 2 or 6 h post stroke, reduced the percentage of foot faults while traversing the ledge-beam test at 24 and 70 h post-stroke (n = 7–11, #*P* < 0.05, ##*P* < 0.01, *P* < 0.0001 vs time-matched sham, *P < 0.05, ***P < 0.001, ****P < 0.0001 vs. time-matched vehicle; analysed with a two-way repeated-measures ANOVA followed by a Tukey’s test). All data is represented as mean ± SEM.

Treatment with 0.1 or 1 nmol HFI419 commencing at 2 h post stroke, and with subsequent doses at 24, 48 and 70 h post-stroke, significantly reduced striatal (19.62 ± 3.18 mm^3^, *P* < 0.001, and 20.45 ± 2.56 mm^3^, *P* < 0.001, respectively) and cortical infarct volume (51.51 ± 10.46 mm^3^, *P* < 0.001 and 60.81 ± 8.12 mm^3^, respectively; Fig. 1A–C) compared to the vehicle treated controls (striatum: 35.26 ± 2.24 mm^3^, cortical: 128.80 ± 16.38 mm^3^). Additionally, treatment with the structurally distinct IRAP inhibitor SJM164 (1 nmol) at identical time-points also significantly reduced striatal and cortical infarct volume (23.59 ± 2.42 mm^3^, 41.07 ± 17.48 mm^3^, respectively *P* < 0.05 vs. vehicle 35.26 ± 2.24 mm^3^ and 128.80 ± 16.38 mm^3^, respectively). Furthermore, the observed neuroprotective effect was sustained when first treatment of 1 nmol HFI419 was delayed until 6 h post-stroke (total infarct volume; 78.75 ± 11.79 mm^3^ vs. vehicle 126 ± 11.72 mm^3^ *P* < 0.05 Fig. 1A–C).

### IRAP inhibitor treatment following ischemic stroke reduced neurological deficit

The neurological deficit score was assessed at 24 and 70 h post-stroke as a broad measure of neurological function where a higher score indicates a worse outcome. Neurological deficits were significantly reduced in all IRAP inhibitor treatment groups regardless of whether the treatment began at 2 or 6 h post-stroke. It is worth noting that the treatment effect was evident at 24 h after stroke, following just a single dose of either SJM164 or HFI419 and was maintained up to 70 h post-stroke (Fig. 1D).

### IRAP inhibitor treatment following ischemic stroke improved motor deficits

In addition to neurological deficit, at 24 and 70 h following stroke, motor deficits were determined by measuring the number of contralateral hindlimb foot faults using the tapered ledge beam test and a rotarod assessment. Treatment with 1 nmol HFI419 beginning at 6 h post stroke significantly improved motor performance on the rotarod compared to the vehicle group as assessed at 70 h post-stroke (4.33 ± 0.64 vehicle vs 6.71 ± 0.62 1 nmol HFI419, *P* < 0.05, Fig. 1E). Treatment with an IRAP inhibitor beginning at 2 h post stroke significantly reduced the percentage of foot faults measured at 24 h after the ischaemic insult (0.1 nmol HFI41 18.70 ± 4.20%; 1 nmol HFI419 22.61 ± 7.65%; 1 nmol SJM164 31 ± 5.47%, *P* < 0.001) compared to vehicle control treatment (71.72 ± 7.91%; Fig. 1F). Additionally, the treatment effect was not diminished when the first dose was delayed to 6 h post-stroke, such that animals receiving HFI419 still performed better than the vehicle group (15.40 ± 5.06% vs 41.85 ± 11.22% respectively, *P* < 0.001). Remarkably, similar to the neurological deficit assessment, a treatment effect was evident as early as 24 h post stroke following only a single dose of IRAP inhibitor that was delivered at either 2 or 6 h after the onset of ischemia. Furthermore, this neuroprotection persisted such that at 70 h post-stroke all groups receiving either SMJ164 or HFI419 performed better than the vehicle treated control animals, regardless of when treatment was commenced.

### IRAP inhibitor treatment enhanced neuronal survival in the cortical ischaemic core

**Figure 2:**
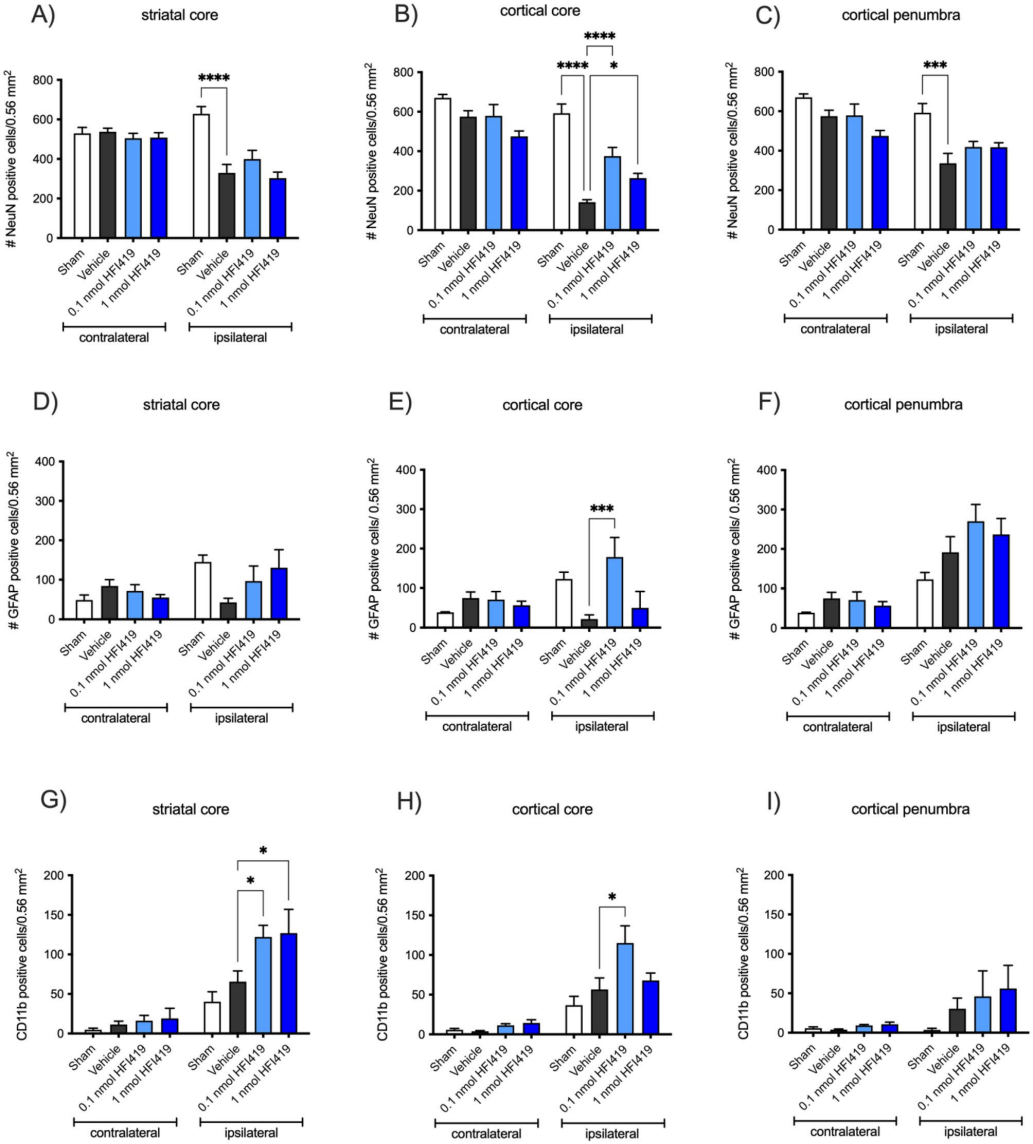
Inhibition of IRAP beginning at 2 h post-stroke attenuated cortical neuronal cell death and increased microglia and astrocyte cell counts within the ischaemic core. Immunohistochemistry (IHC) using a neuronal marker NeuN (**A**–**C**) to visualise neuronal bodies within the striatal and cortical core as well as the cortical penumbra at 72 h post-stroke revealed that treatment with 0.1 or 1 nmol HFI419 beginning at 2 h, and then at 24, 48 and 70 h post-stroke, attenuated neuronal cell death within the cortical core. IHC using the astrocyte specific marker GFAP (**D**–**E**), revealed an increased GFAP positive cell count within the cortical core following treatment with 0.1 nmol HFI419. Lastly, IHC using the microglial marker CD11b (**F**–**I**) revealed an increased CD11b cell count within the striatal and cortical core following treatment with 0.1 or 1 nmol HFI419. The data is represented as mean ± SEM and analysed using a two-way ANOVA followed by a Dunnett’s test where *P < 0.05 and ***P < 0.001 (n = 4–9).

Treatment with an IRAP inhibitor reduced infarct volume and consequently improved neurological and motor function. However, to determine whether additional neuronal survival within the striatal or cortical core also contributed to improved functional outcome, surviving neurons at 72 h post-stroke were visualised and counted after immunostaining with the nuclear marker of neurones, NeuN (Fig. 2A–C). Stroke resulted in a significantly reduced number of NeuN positive cells in the striatum and cortical core regions. Treatment with either 0.1 or 1 nmol HFI419 beginning at 2 h post-stroke attenuated this loss of NeuN positive staining in the infarcted cortex (375.33 ± 43.62 cells per 0.54 mm^2^, *P* < 0.0001 and 263.67 ± 24.18 cells per 0.54 mm^2^, *P* < 0.05, respectively) compared to vehicle treated controls (141.43 ± 12.82 cells per 0.54 mm^2^, Fig. 2B). Within the cortical penumbra, there was a significant reduction of NeuN positive staining in the vehicle control compared to sham however we did not detect any difference with any IRAP inhibitor treatment group compared to vehicle (Fig. 2C). When delaying the first dose of HFI419 to 6 h post-stroke, despite the reduction of infarct volume with treatment, there was no evidence of additional neuronal preservation within any brain region compared to the vehicle treated animals (Supplementary Fig. 2A–C).

### IRAP expression was upregulated in CD11b positive cells and astrocytes in the injured ischaemic brain

**Figure 3:**
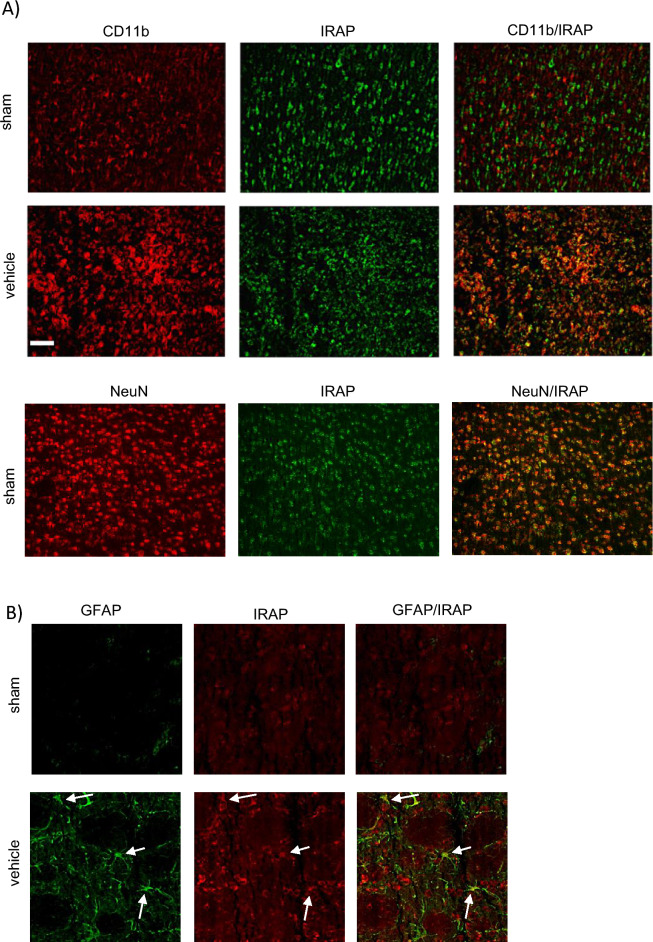
Upregulation of IRAP expression in CD11b and GFAP positive cells following stroke. Representative immunofluorescent images demonstrating (**A**) upregulation of IRAP in activated CD11b positive cells within the stroked cortical core and (**B**) and in GFAP positive cells within the stroked striatum (white arrows) at 72 h post-stroke. (**A**) The white scale bar represents 100 µm with images in panel (**A**) and (**B**) captured at equal magnification.

CD11b positive cells in the ischaemic regions were found to express IRAP at 72 h post-stroke, as evident by co-localisation with IRAP immunostaining (Fig. 3A), unlike in quiescent brain where there is minimal CD11b/IRAP colocalization. Interestingly, IRAP inhibitor treatment beginning at 2 h post-stroke significantly increased CD11b within the striatal and cortical core with 0.1 nmol HFI419 (striatum; 122.11 ± 14.72 cells per 0.54 mm^2^, *P* < 0.01, cortex; 115.11 ± 21.63 cells per 0.54 mm^2^, *P* < 0.01) and striatal core with 1 nmol of HFI419 (126.83 ± 30.13 cells per 0.54 mm^2^, *P* < 0.05) compared to sham control (striatum; 40.33 ± 12.3 cells per 0.54 mm^2^, cortex; 36.83 ± 11.10 cells per 0.54 mm^2^ Fig. 2G–H). There was no difference in CD11b within the cortical penumbra between vehicle or inhibitor treated groups (Fig. 2I). In addition, a small population of activated astrocytes were shown to express IRAP following stroke as demonstrated by GFAP and IRAP colocalization (Fig. 3B). At 72 h following stroke, the critically injured ischaemic core demonstrated a reduced GFAP cell count compared to sham however animals treated with 0.1 nmol of HFI419 beginning at 2 h post-stroke demonstrated a greater number of activated astrocytes within the cortical core (178.62 ± 49.62 cells per 0.54 mm^2^, *P* < 0.01) compared to the vehicle control (21.81 ± 10.78 cells per 0.54 mm^2^, Fig. 2E). GFAP positive cell counts in the striatal core and cortical penumbra did not differ between treatment groups (Fig. 2D and F). When the first dose of HFI419 was delayed to 6 h post stroke, treatment significantly increased CD11b counts in the cortical penumbra (Supplementary Fig. 2I) however no differences were detected in the number of GFAP positive cells between the drug treated and vehicle treated groups in any brain region (Supplementary Fig. 2D–F).

### Treatment with HFI419 2 h post stroke upregulated IL-6 and CCL2

**Figure 4:**
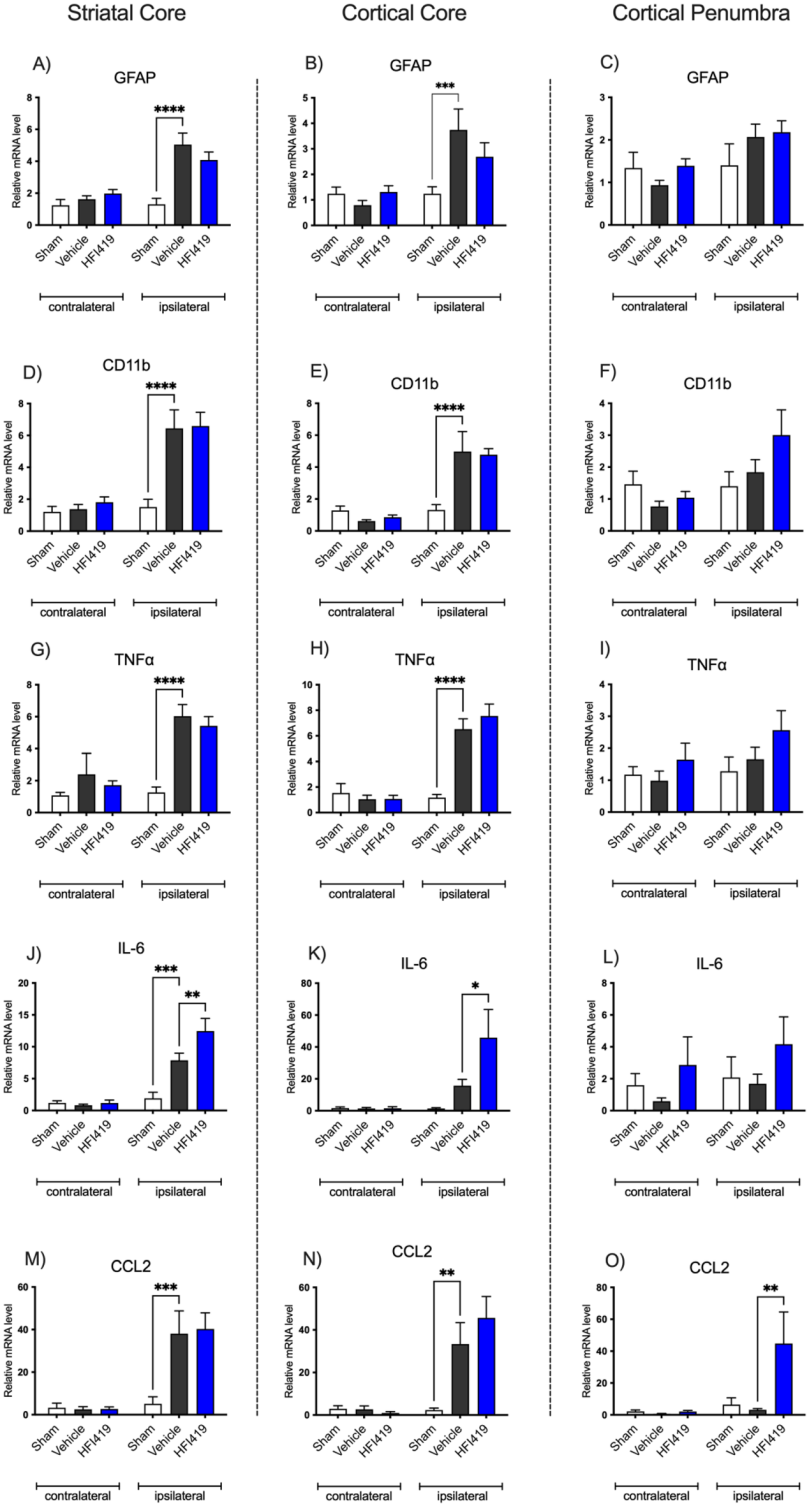
IRAP inhibition beginning at 2 h post-stroke upregulated IL-6 and CCL2 24 h. Following treatment with 0.1 nmol HFI419 2 h post-stroke, the gene expression of inflammatory mediators was determined in the ipsilateral and contralateral striatum, cortical core, and cortical penumbra at 24 h post-stroke. Following treatment with an IRAP inhibitor at 2, 24, 48 and 70 h post-stroke, (**J** & **K**) IL-6 was upregulated in the striatal and cortical core compared to vehicle control. In the cortical penumbra, treatment with an IRAP inhibitor upregulated (**M**–**O**) CCL2 compared to vehicle treatment. The data is represented as mean ± SEM and analysed using a two-way ANOVA followed by a Dunnett’s test (*P < 0.05, **P < 0.01, ***P < 0.001, ****P < 0.0001; n = 5–6).

IRAP was upregulated in activated astrocytes and CD11b positive immune cells in the post-ischaemic brain. To further delineate the neuroinflammatory role of IRAP post-stroke, cytokine and chemokine profiles were examined at 24 h post-stroke (Supplementary Table 1). In contrast to our immunohistochemical analysis of the brains at 72 h post stroke, at 24 h post stroke, the expression of CD11b and GFAP genes were upregulated in the striatal and cortical core but not in the penumbra in the ischaemic hemisphere of all groups (Fig. 4A–F). Furthermore, the gene expression of the inflammatory cytokines, TNF⍺ and IL-6, as well as the chemokine CCL2, were upregulated in the striatal and cortical ischaemic core (Fig. 4G–O). Treatment with HFI419 potentiated the upregulation of IL-6 in the cortical and striatal core (Fig. 4J–K) and upregulated CCL2 in the cortical penumbra (Fig. 4O) compared to vehicle control. When brain tissue was assessed at 72 h post stroke, there was no treatment effect evident on any of the cytokine or chemokine of interest (Supplementary Table 2).

### Treatment with HFI419 attenuated stroke-induced increases in forebrain volume

**Figure 5:**
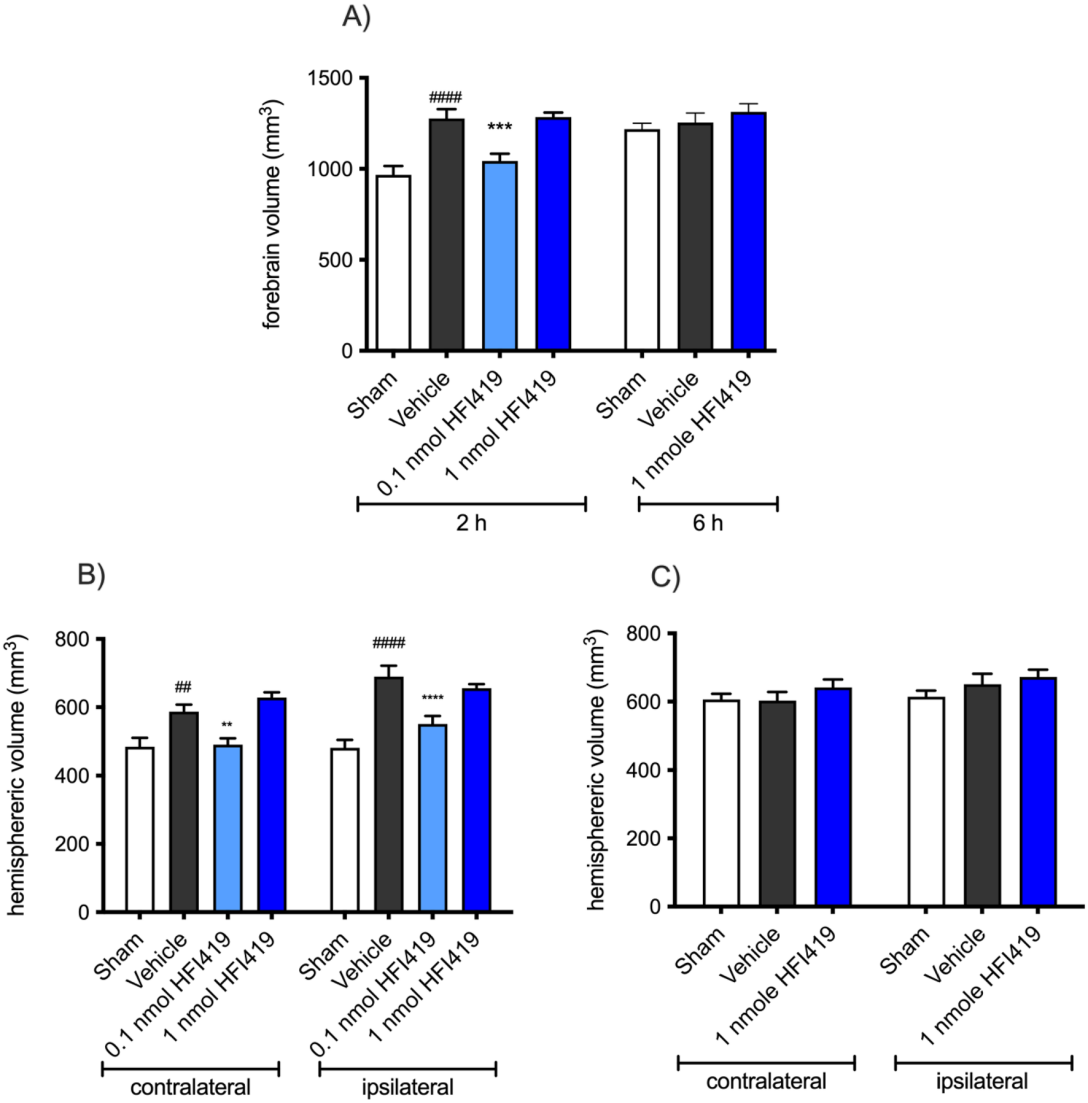
Treatment with an IRAP inhibitor reduced forebrain volume following stroke. (**A**) At 72 h post-stroke, (**A** & **B**) treatment with 0.1 or 1 nmol HFI419 beginning at 2 h post-stroke significantly reduced total forebrain volume. (**C**) There was no change in oedema when treatment was delayed to 6 h post-stroke. The data is represented as mean ± SEM (**A**; ####*P* < 0.0001 vs sham, *P < 0.05 vs vehicle, ***P < 0.001 vs vehicle, one-way ANOVA, n = 5–10) (**B** and **C**; ##*P* < 0.01, #### *P* < 0.0001 vs sham matched hemisphere, **P < 0.01, ****P < 0.0001 vs vehicle matched hemisphere, two-way ANOVA, followed by a Dunnett’s test, n = 5–10).

Vasopressin is a physiological substrate of IRAP^45^ and can exacerbate cerebral oedema^46^. To assess if IRAP inhibition post-stroke influenced cerebral oedema, forebrain volumes were assessed. At 72 h following stroke, total forebrain volume of the vehicle treated group (1276.00 ± 51.02 mm^3^) was significantly greater than sham (966.50 ± 49.20 mm^3^; *P* < 0.001; Fig. 5A). Treatment with 0.1 nmol HFI419 significantly attenuated the stroke-induced increase in forebrain volume (1043 ± 40.38 mm^3^ vs vehicle, *P* < 0.01). Surprisingly, treatment with 1 nmol HFI419 did not show the same level of protection against oedema as the animals receiving the lower dose of 0.1 nmol HFI419 (Fig. 5B). No effect of oedema was evident in animals treated 6 h post-stroke (Fig. 5C).

### Neuroprotection mediated by IRAP inhibition was independent of regulation of systolic blood pressure, vascular reactivity or microvascular density

**Figure 6:**
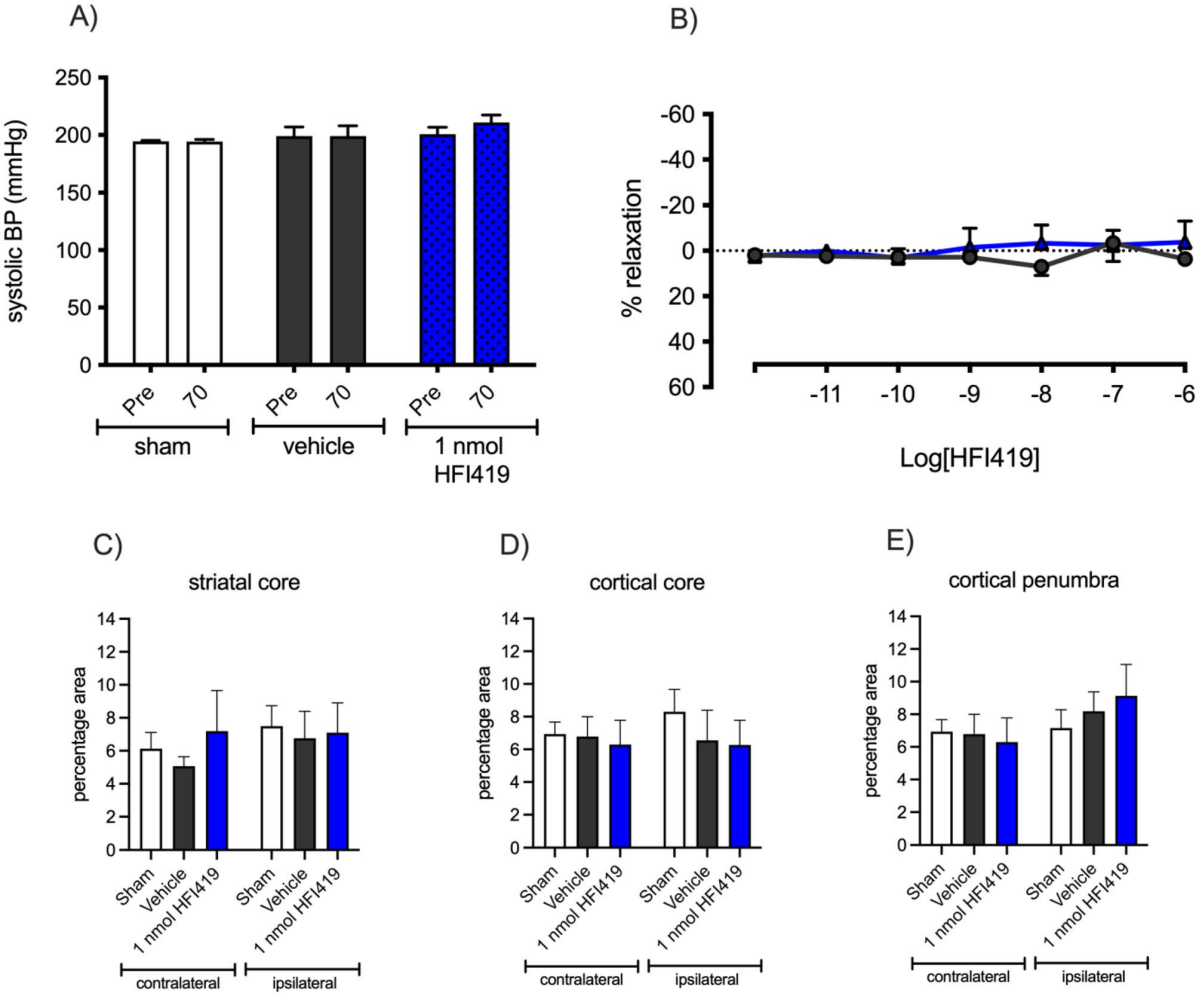
Neuroprotection mediated by IRAP inhibition was independent of changes in blood pressure, vascular reactivity and vascular density. (**A**) Systolic blood pressure determined pre stroke and at 70 h post stroke was not different between sham operated, vehicle and HFI419 (1 nmol) treated animals. (**B**) Myograph preparations of the SHR basilar artery indicated no change in vascular reactivity in response to increasing concentrations of HFI419 (n = 3–5; data represented by mean ± SEM). Comparison of GLUT1 immunopositively stained cell area within the striatal core (**C**), cortical core (**D**) and cortical penumbra (**E**) revealed no differences with either stroke or treatment with an IRAP inhibitor (n = 4–9, data represented by mean ± SEM).

The SHR is a hypertensive animal model and to determine if any neuroprotective effect of IRAP inhibition was secondary to hypertension management, systolic blood pressure was assessed via tail cuff prior to stroke and at 72 h post-stroke. Stroke did not alter blood pressure of the rats (Fig. 6A). Furthermore, central administration of HFI419 post-stroke had no apparent blood pressure lowering effects (Fig. 6A). In addition, in vitro analysis of vascular reactivity in a pre-constricted isolated basilar artery for a naïve SHR using a wire myograph, indicated that increasing doses of HFI419 failed to elicit any vascular relaxation responses (Fig. 6B). Finally, immunohistochemistry visualising the cerebral blood vessel marker GLUT 1 within the cortex and striatum was performed to determine if improvements with HFI419 treatment following stroke was as a result of angiogenesis. In all treatment groups, no differences in GLUT 1 were detected within the cortex and striatum suggesting that there was no effect of stroke or drug treatment on cerebral microvascular density (Fig. 6C–E).

## Discussion

The major findings of the current study are that treatment with an IRAP inhibitor 2 h after stroke protected against ischemic damage resulting in improved functional outcomes. We also found that this neuroprotection was not diminished even when the first administration of the IRAP inhibitor was delayed to 6 h post stroke. To date, apart from fibrinolytic therapies, all drug candidates for the treatment of stroke have demonstrated poor clinical translation, despite their proven efficacy in preclinical models^47,48^. This highlights an ongoing need for novel therapies to be developed to improve stroke care either as an adjuvant therapy to current clinical practice or as a standalone monotherapy. We have previously shown that global IRAP gene deletion was neuroprotective following transient occlusion of the MCA in mice^11^ congruous with an earlier report demonstrating that intracarotid administration of angiotensin IV (Ang IV), a competitive albeit non-selective inhibitor of IRAP^49^, significantly reduced infarct volume, attenuated neurological deficits and increased survival rate in a rat model of embolic stroke (Faure et al. 2006). Despite IRAP being identified as the Ang IV binding site, Ang IV also binds to various other proteins including the angiotensin II AT1 and AT2 receptors and aminopeptidase N (Yamamoto et al. 2010). This promiscuity naturally gave rise to uncertainty as to whether IRAP was, in fact, the target responsible for the neuroprotection induced by exogenous administration of Ang IV. We now provide proof-of-principle that treatment with two different classes of small molecular weight selective IRAP inhibitors, HFI419 or SJM164, provided protection in a conscious model of stroke in the hypertensive rat. While the mechanism of IRAP inhibition remains to be fully elucidated, IRAP was upregulated in activated astrocytes and CD11b positive cells following stroke suggesting that IRAP plays an immunomodulatory role in the ischemic brain.

Both HFI419 and SJM164 significantly reduced cortical infarct volume at 72 h after stroke, even when the therapeutic window was extended to 6 h. Impressively, when treatment was commenced at 2 h post-stroke, the neuroprotective effect of IRAP inhibition extended to the striatum, which is a region of the brain that is traditionally thought be refractory to therapeutic intervention^50^. Consistent with a significant reduction in infarct volume, treatment with HFI419 attenuated the physical symptoms of stroke, as demonstrated by improved performance on the tapered ledge-beam test and the rotarod as well as a reduction in the neurological deficit score. Impressively, these benefits were evident as early as 24 h post-stroke, following administration of just a single dose of either HFI419 or SJM164. This improvement in the physical symptoms of stroke, is most likely a reflection of the reduction of infarct volume and preservation of penumbral tissue. However, with early administration of treatment at 2 h post-ischemia, the IRAP inhibitor treated brains demonstrated increased neuronal survival within the infarcted cortical core. More importantly, we found that extending the interventional therapeutic window whereby the first dose of IRAP inhibitor was received at 6 h post-stroke, neuroprotection was still clearly evident by way of improvements in histological and behavioural assessments. From a clinical perspective, the therapeutic efficacy following delayed delivery of the first drug dose broadens potential patient candidacy, particularly as the symptom onset-to-hospital arrival times have remained largely unchanged over the last two decades with the majority of stroke patients presenting to hospital within 6 h from symptom onset^51,52^.

Immunologically, IRAP has been shown to be critical in major histocompatibility complex cross presentation^53^ as well as T-cell receptor signalling^54^, and in this study we reveal for the first time that IRAP was upregulated in both activated astrocytes and CD11b positive CNS immune cells at 72 h post-stroke, which is suggestive of an inflammatory role for IRAP in the post ischaemic brain. Our finding is congruent with earlier studies that demonstrated an upregulation of IRAP in activated in peripherally activated macrophages following interferon gamma or lipopolysaccharide-induced inflammation (Nikolaou et al. 2014). In the current findings, treatment with 0.1 nmol HFI419 beginning at 2 h post-stroke increased recruitment of astrocytes and CD11b positive cells to the stroke-induced site of injury, both of which are critical for post-stroke neuronal repair and remodelling. Although cellular infiltration was not evident when treatment with an IRAP inhibitor commenced at 6 h post-stroke, delayed treatment still reduced infarct volume and improved functional outcome suggesting that the benefits of IRAP inhibition are multifactorial and may vary depending on the temporality of the ischemic cascade.

To further investigate the inflammatory role of IRAP, pro- and anti-inflammatory cytokine and chemokine gene expression were quantified at 24 and 72 h post-stroke where treatment with 0.1 nmol HFI419 began at 2 h post-stroke. At 24 h post-stroke, CCL2 and IL-6 were significantly upregulated following a single dose of HFI419. We first observed the benefits of IRAP inhibition at 24 h post-stroke following a single dose, with improved performance on the ledge-beam test and reduced neurological deficits. Taken together, these findings suggest that IL-6 and CCL2 may play a role in mediating early neurological protection following IRAP inhibition.

Certainly, peak IL-6 serum levels post-stroke correlated with the extent of infarct size and neurological deficit^55^ however whether or not this is causative is equivocal^56^ particularly given that upregulation of IL-6 is protective against NMDA-mediated excitotoxicity^57^. Experimentally, IL-6 is neuroprotective in in vivo stroke models whereby intracerebroventricular injection of recombinant IL-6 reduced infarct volume and improved neurological deficits^58^ and blockade of IL-6 signalling with intraperitoneal injections of an IL-6 receptor antibody exacerbated ischaemic brain injury^59^. Additionally, IL-6 is essential for promoting post stroke angiogenesis^60^ as well as neurogenesis and long-term functional recovery^61^. In the current study, HFI419 treatment resulted in IL-6 upregulation in both the cortical and striatal core regions of damage consistent with a neuroprotective role for IL-6 as infarct volume was significantly reduced in both of these regions. Despite these findings, there is still ambivalence surrounding the role IL-6 following stroke as global IL-6 gene deletion demonstrate comparable infarct volume to controls with reductions in body temperature^62^. However, if body temperature is controlled for by external warming, global IL-6 deletion resulted in a larger infarct volume and worse neurological outcome favouring an overall neuroprotective role of IL-6 independent of its pyrogenic properties^63^.

The monocyte chemoattractant protein-1 (MCP1/CCL2) was upregulated in the penumbra with IRAP inhibitor treatment following stroke. CCL2 is a potent monocyte chemoattractant that mediates its effects through its receptor CCR2^64^. Acutely following stroke, CCR2 KO mice exhibited larger infarct volume, cerebral oedema, worse neurological function as well as increased blood brain barrier permeability^65,66^. However, long-term post-stroke follow-up studies in these mice demonstrated increased mortality rates and abolished long-term neurological and functional recovery^67,68^. Moreover, aberrant CCL2/CCR2 signalling resulted in a larger infarct volume^69^, a higher incidence of haemorrhagic transformation^70^ and reduced angiogenesis^71^. The benefits of CCL2 and CCR2 are likely related to the recruitment of Ly6C^hi^ peripheral monocytes and their ability to differentiate into the anti-inflammatory/reparative M2 phenotype ^69,71,72^. In our study, HFI419 treatment upregulated CCL2 expression in the cortical penumbra and unsurprisingly this was associated with a regionally matched increase in CD11b cell counts. Despite an elevated CCL2 with drug treatment, IRAP inhibition with a low dose of HFI419 reduced stroke related increased forebrain volume suggestive of reduced cerebral oedema. The reduction of forebrain volume likely represents a dose response effect of HFI419 given that the higher dose of HFI419 did not reduce oedema despite reducing infarct volume. Indeed, IRAP is expressed in peripheral macrophages with greater expression in M1 macrophages, however inhibition of the catalytic domain with Ang IV did not reduce pro-inflammatory cytokines^14^, a finding consistent with the current study. Nevertheless, acutely following stroke, recruited microglia are predominantly polarised to the M2 phenotype^73^. Although further studies are required to confirm, it is plausible that IRAP inhibition post-stroke increases monocyte recruitment through elevated CCL2 and therefore promotes M2 polarisation, or that direct IRAP inhibition preferentially differentiates monocyte/microglia M2 phenotype via an alternative mechanism. Lastly, in addition to monocyte recruitment, CCL2 and its receptor CCR2 play an integral role in endogenous neuroblast differentiation and migration to injured tissue^74,75^ as well as the homing of peripherally delivered stem cell therapy following stroke^74,76,77^. Interestingly, IRAP is highly expressed in the subventricular zone^78^ and postulated to play a role in neuronal development^49,79^ and although this mechanism requires further investigation, it may hold significant implications for long-term stroke recovery.

In addition to immunomodulation, augmentation of collateral blood flow may be another potential contributor to the therapeutic benefits of post-stroke IRAP inhibition. Inhibition of IRAP, in both the IRAP KO mice or rats treated with the IRAP inhibitor Ang IV, have been shown to enhance collateral blood flow post-stroke^11,80^. Consistent with this, IRAP inhibitors delivered locally to the pulmonary^81^, renal^82^ and cerebral vasculature^83,84^ have been demonstrated to promote dilatation and increased blood flow. In these studies, the dilatory effects are inhibited in the presence of L-NAME and therefore likely mediated via a nitric oxide dependent pathway possibly through the increased availability of IRAP substrates such as the NO-dependent vasodilator bradykinin^85^. We were not able to directly measure cerebral blood flow following MCA occlusion in the current study, a limitation of the conscious stroke model, however HFI419 did not elicit any vasoactive responses in the isolated healthy basilar artery, which suggests that the protection we have demonstrated is unlikely to have a vascular component. Furthermore, we did not detect increased endothelial NOS expression following treatment with HFI419 at either 24 or 72 h post-stroke nor was there a difference in cerebral microvascular density at 72 h post-stroke. Nevertheless, our findings do not exclude the possibility of an acute dilatory effect in an injured vessel following IRAP inhibition, as improved collateral blood flow in previous stroke studies was demonstrated either during the transient occlusion period^11^, or within minutes after stroke and drug administration^80^ in the dysfunctional vessel^86^. Additionally, considering long term IRAP inhibition reverses chronic vessel dysfunction^87^ and that IRAP inhibition improves learning and memory^21,22,24,28,29^ as well as reducing seizure susceptibility^30,88^, there is strong rationale to further explore the effects of chronic IRAP inhibition following stroke in the context of preventing complicating vascular and neurological sequelae.

Despite numerous clinical candidates being effectively trialled in preclinical stroke models, translation to human clinical trials has been largely unsuccessful in part due to limitations in study design. The current study design attempts to address some of these shortcomings by the choice of the model, the ET-1 model of stroke in a conscious animal which (i) eliminates the confounding variable of anaesthesia^89^, (ii) blood flow pattern that closely mimic what is seen in humans and (iii) includes hypertension as a co-morbidity. Moreover, administration of the neuroprotective agent was delayed to 6 h after the induction of stroke, which is a clinically feasible timeline for treatment delivery. One limitation of the current study is the central route of drug administration. An intracerebroventricular mode of delivery was chosen to ensure adequate central access of the IRAP inhibitors in this proof of principle study. As such, the impact of the BBB on drug delivery was not considered. Nevertheless, approximately 20% of intraperitoneal injected HFI419 reaches the brain 5 min post-injection in the healthy Sprague Dawley^90^. Moreover, the BBB is known to be compromised following ischemic stroke^91^ and therefore the central nervous system bioavailability of peripherally delivered IRAP inhibitor following stroke is likely greater in the injured brain. The compounds used in the present study, HFI419 and SJM164, are small molecular weight, specific inhibitors of IRAP. In contrast to Ang IV or other peptide inhibitors, they demonstrate superior selectivity for IRAP and a reduced susceptibility to systemic degradation^28^ which is favourable for a peripheral and clinically relevant route of administration.

Currently, the only pharmacological intervention following ischemic stroke is tPA, however this therapy is only accessible to a limited number of patients and offers no additional therapeutic benefit beyond the restoration of cerebral blood flow. This underscores the urgent need for novel therapeutics with a wider therapeutic window. We have shown, for the first time, that pharmacological inhibition of IRAP via central administration of two distinct classes of small molecular weight IRAP inhibitors significantly improved morphological and functional outcomes following stroke in the SHR, even when treatment was delayed to 6 h post stroke. Although further research is required to elucidate the cellular mechanism responsible for the neuroprotection demonstrated, our current understanding suggests that there are likely various components to the therapeutic benefit of IRAP inhibition, including modulation of the innate immune response and preservation of neuronal survival ultimately leading to improved motor and neurological function. The findings of this study provide compelling evidence that inhibition of IRAP significantly improves ischemic stroke outcomes and identifies IRAP as a potential target for the development of novel neuroprotective agents for the treatment of ischaemic stroke.

### Supplementary Information

Supplementary Figure 1.

Supplementary Figure 2.

Supplementary Table 1.

Supplementary Table 2.

## Data Availability

The data that support the findings of this study are available in the methods and/or supplementary material of this article.
