# Supplementary material for: Inhibition of insulin-regulated aminopeptidase confers neuroprotection in a conscious model of ischemic stroke
Source: Sci Rep. 2023 Nov 13;13:19722. doi: 10.1038/s41598-023-46072-5 (PMC10643421; doi:10.1038/s41598-023-46072-5)
Supplement: Supplementary file 1 — Supplementary Figure 1. [file 41598_2023_46072_MOESM1_ESM.pptx]

## Slide 1
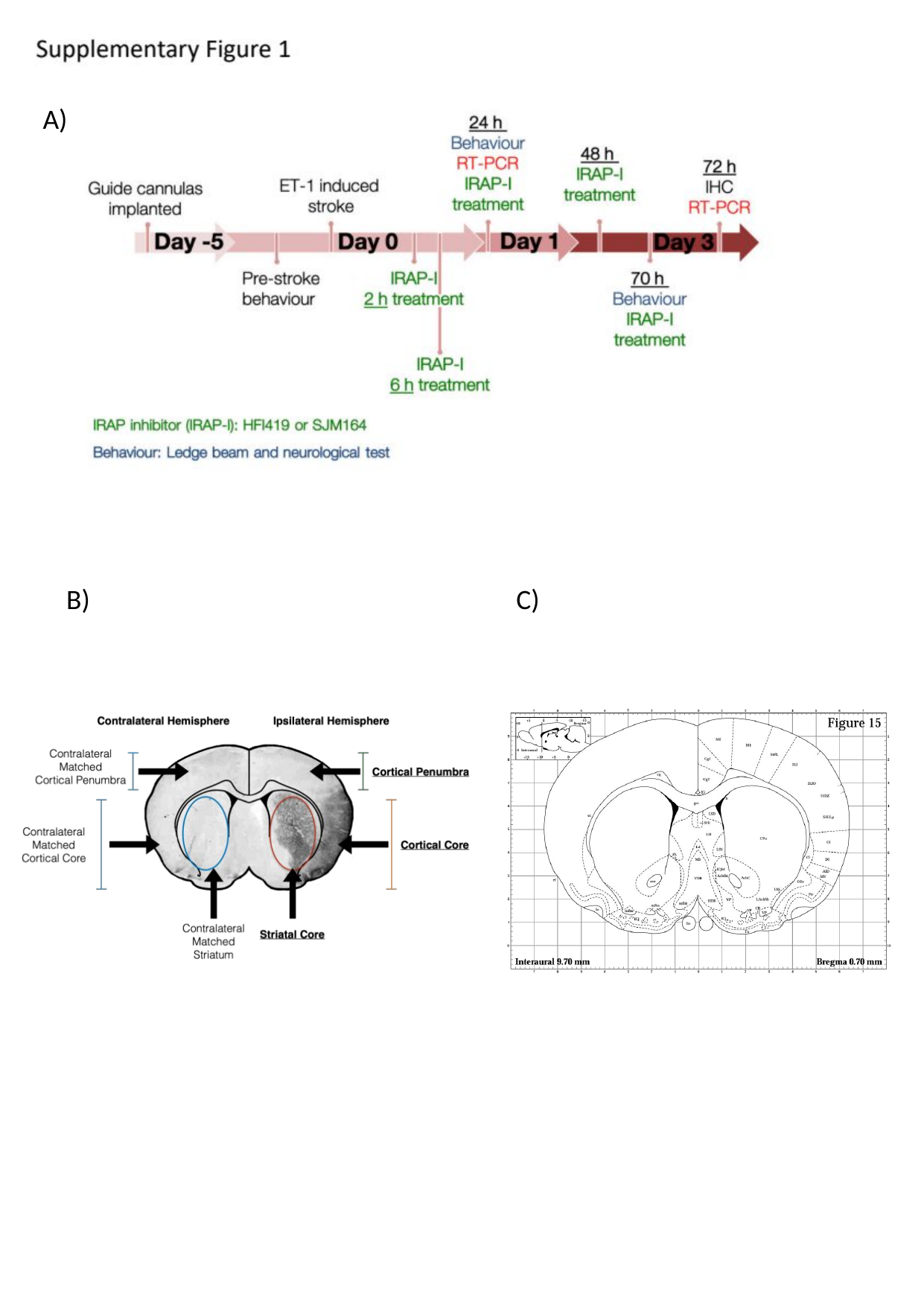

A)
B)
C)

## Slide 2
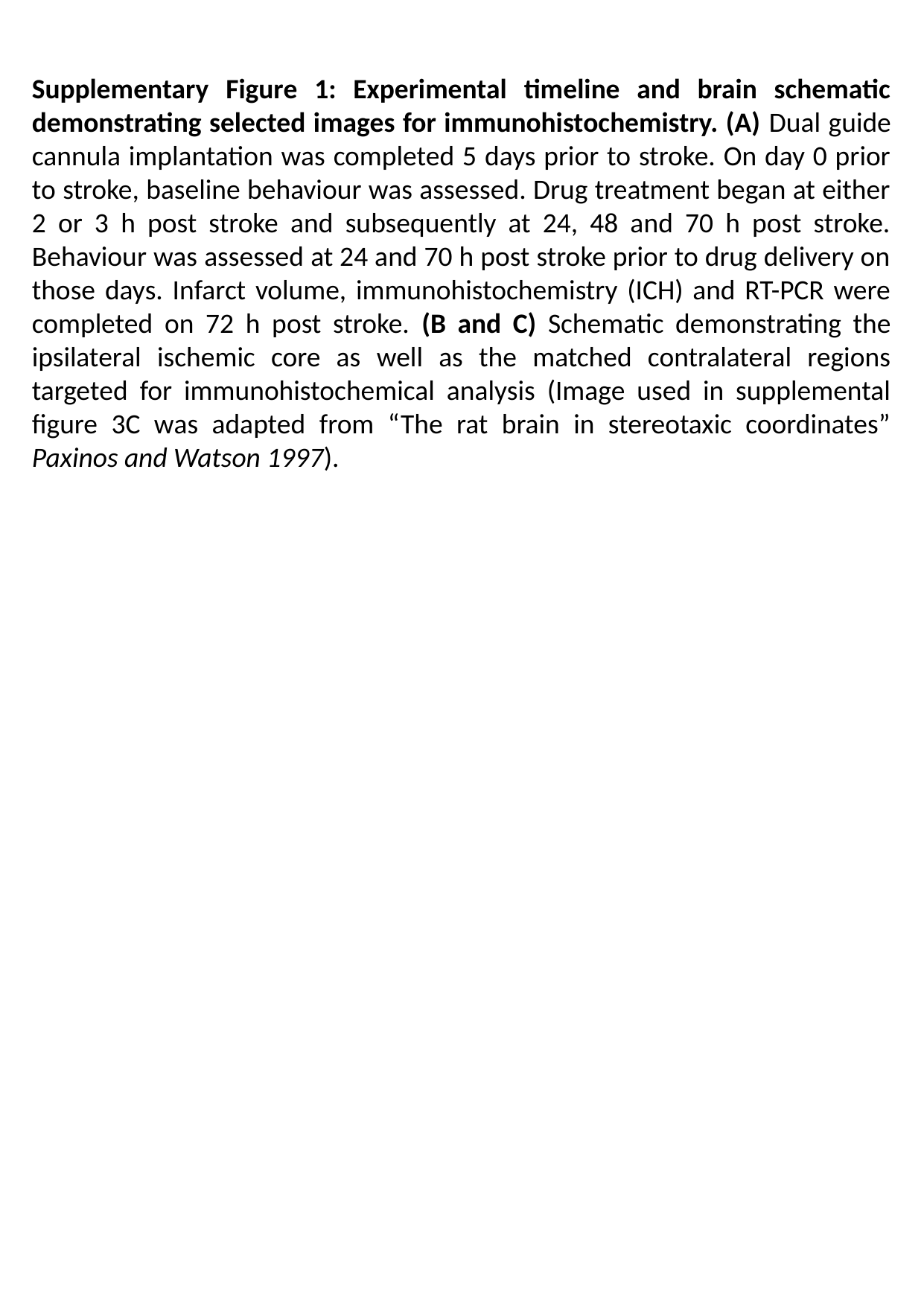

Supplementary Figure 1: Experimental timeline and brain schematic demonstrating selected images for immunohistochemistry. (A) Dual guide cannula implantation was completed 5 days prior to stroke. On day 0 prior to stroke, baseline behaviour was assessed. Drug treatment began at either 2 or 3 h post stroke and subsequently at 24, 48 and 70 h post stroke. Behaviour was assessed at 24 and 70 h post stroke prior to drug delivery on those days. Infarct volume, immunohistochemistry (ICH) and RT-PCR were completed on 72 h post stroke. (B and C) Schematic demonstrating the ipsilateral ischemic core as well as the matched contralateral regions targeted for immunohistochemical analysis (Image used in supplemental figure 3C was adapted from “The rat brain in stereotaxic coordinates” Paxinos and Watson 1997).
