# Supplementary material for: Inhibition of insulin-regulated aminopeptidase confers neuroprotection in a conscious model of ischemic stroke
Source: Sci Rep. 2023 Nov 13;13:19722. doi: 10.1038/s41598-023-46072-5 (PMC10643421; doi:10.1038/s41598-023-46072-5)
Supplement: Supplementary file 2 — Supplementary Figure 2. [file 41598_2023_46072_MOESM2_ESM.pptx]

## Slide 1
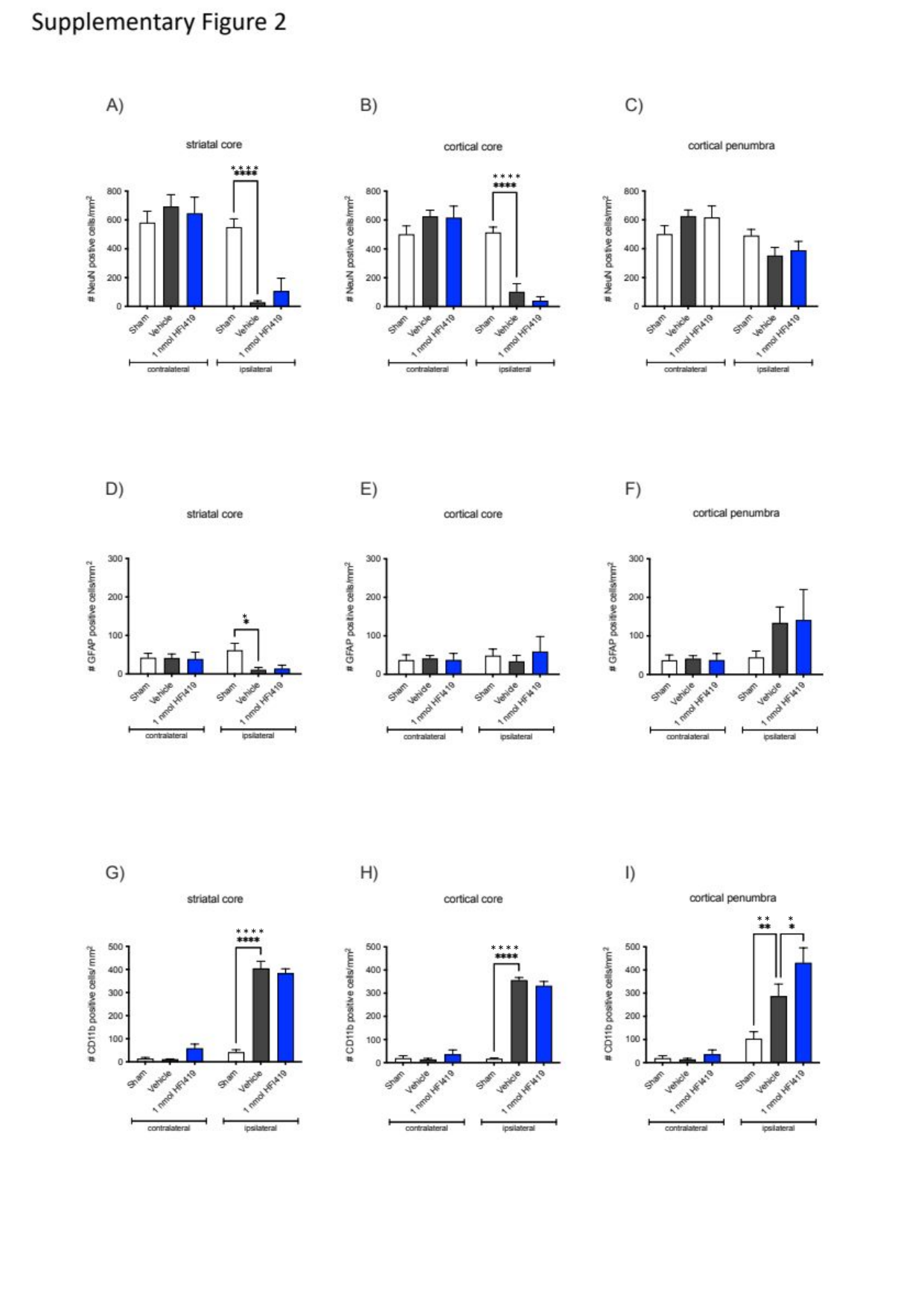

****
****
*
**
*
****
****

## Slide 2
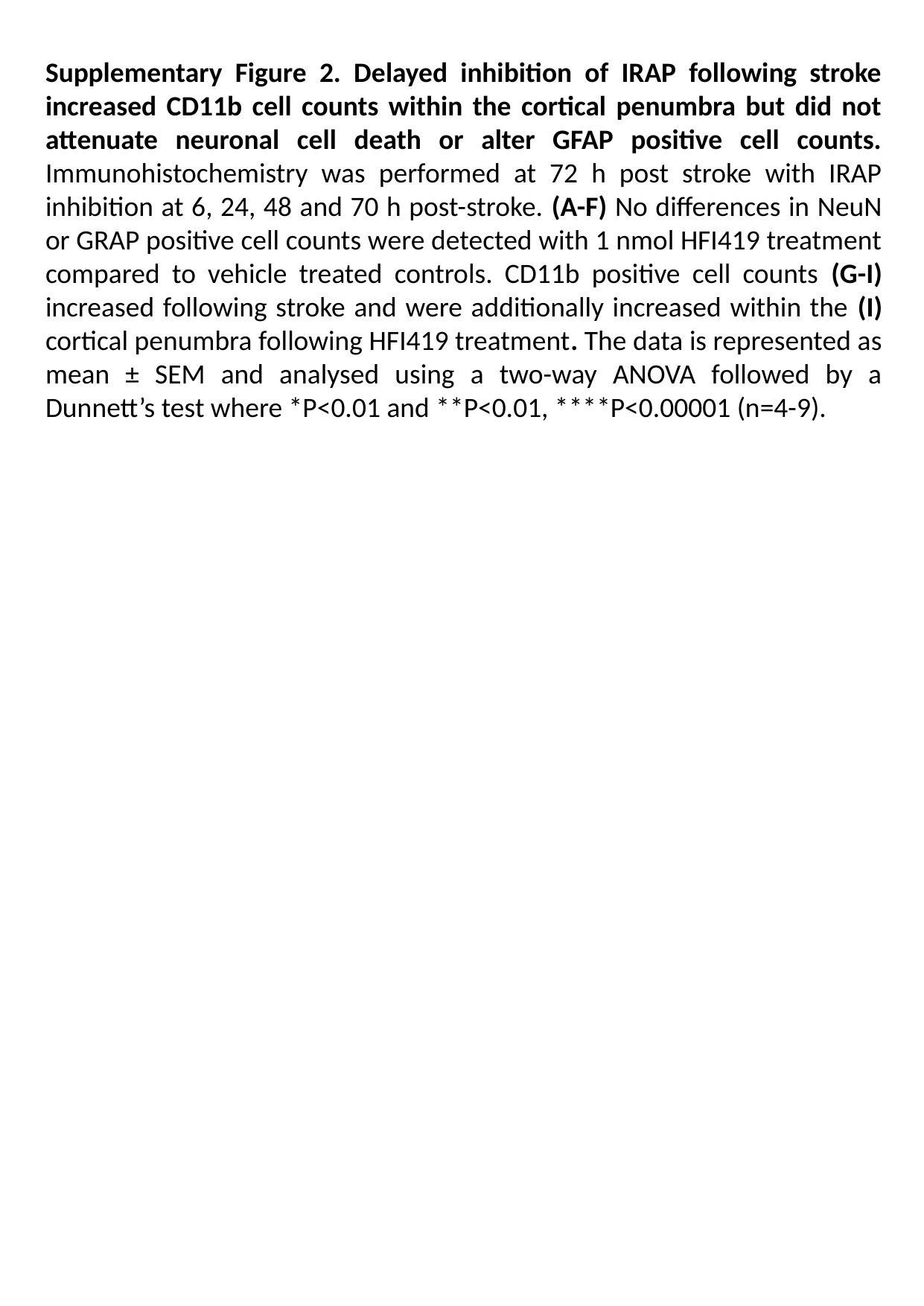

Supplementary Figure 2. Delayed inhibition of IRAP following stroke increased CD11b cell counts within the cortical penumbra but did not attenuate neuronal cell death or alter GFAP positive cell counts. Immunohistochemistry was performed at 72 h post stroke with IRAP inhibition at 6, 24, 48 and 70 h post-stroke. (A-F) No differences in NeuN or GRAP positive cell counts were detected with 1 nmol HFI419 treatment compared to vehicle treated controls. CD11b positive cell counts (G-I) increased following stroke and were additionally increased within the (I) cortical penumbra following HFI419 treatment. The data is represented as mean ± SEM and analysed using a two-way ANOVA followed by a Dunnett’s test where *P<0.01 and **P<0.01, ****P<0.00001 (n=4-9).
