# Supplementary material for: Inhibition of insulin-regulated aminopeptidase confers neuroprotection in a conscious model of ischemic stroke
Source: Sci Rep. 2023 Nov 13;13:19722. doi: 10.1038/s41598-023-46072-5 (PMC10643421; doi:10.1038/s41598-023-46072-5)
Supplement: Supplementary file 4 — Supplementary Table 2. [file 41598_2023_46072_MOESM4_ESM.pptx]

## Slide 1
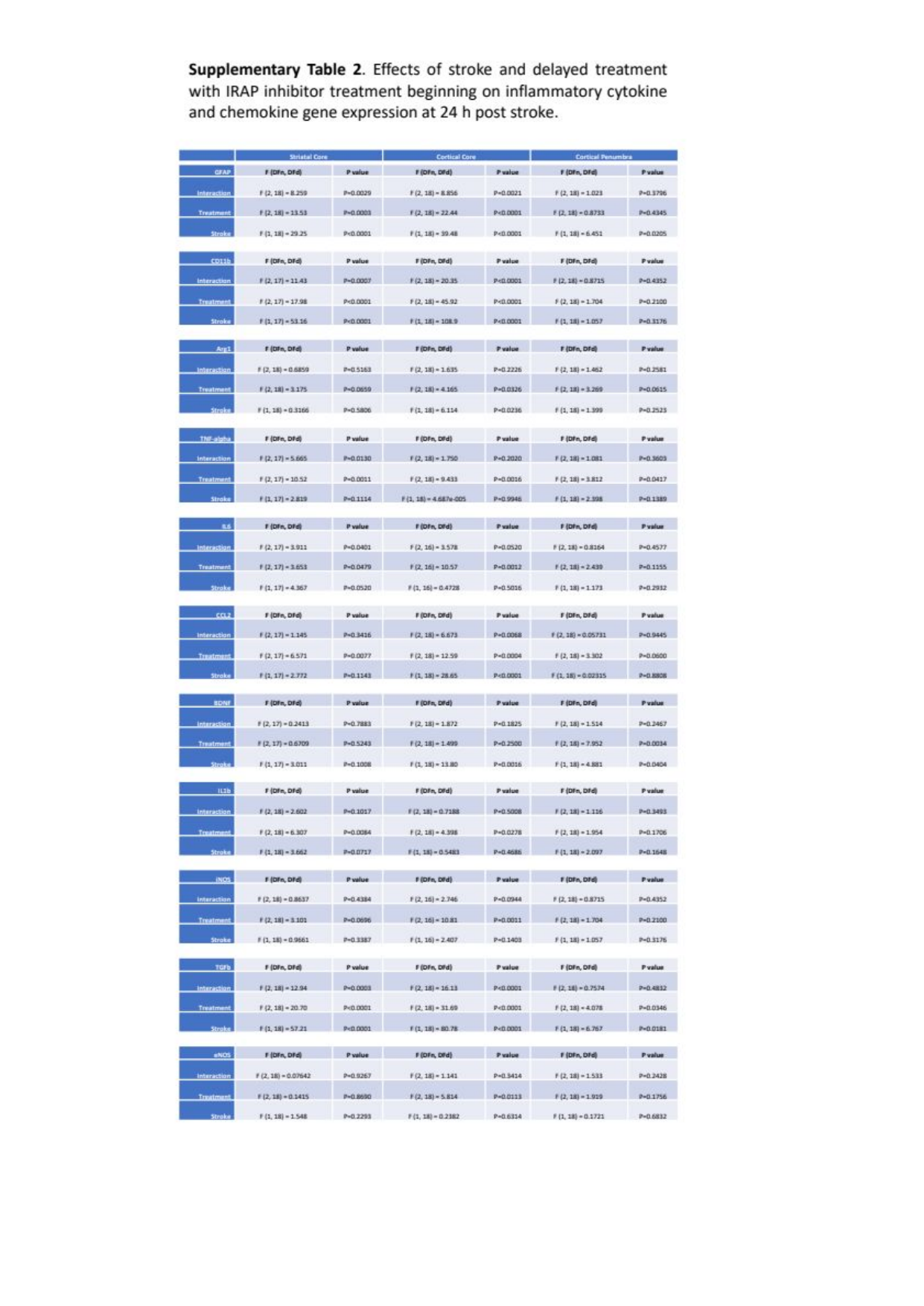

## Slide 2
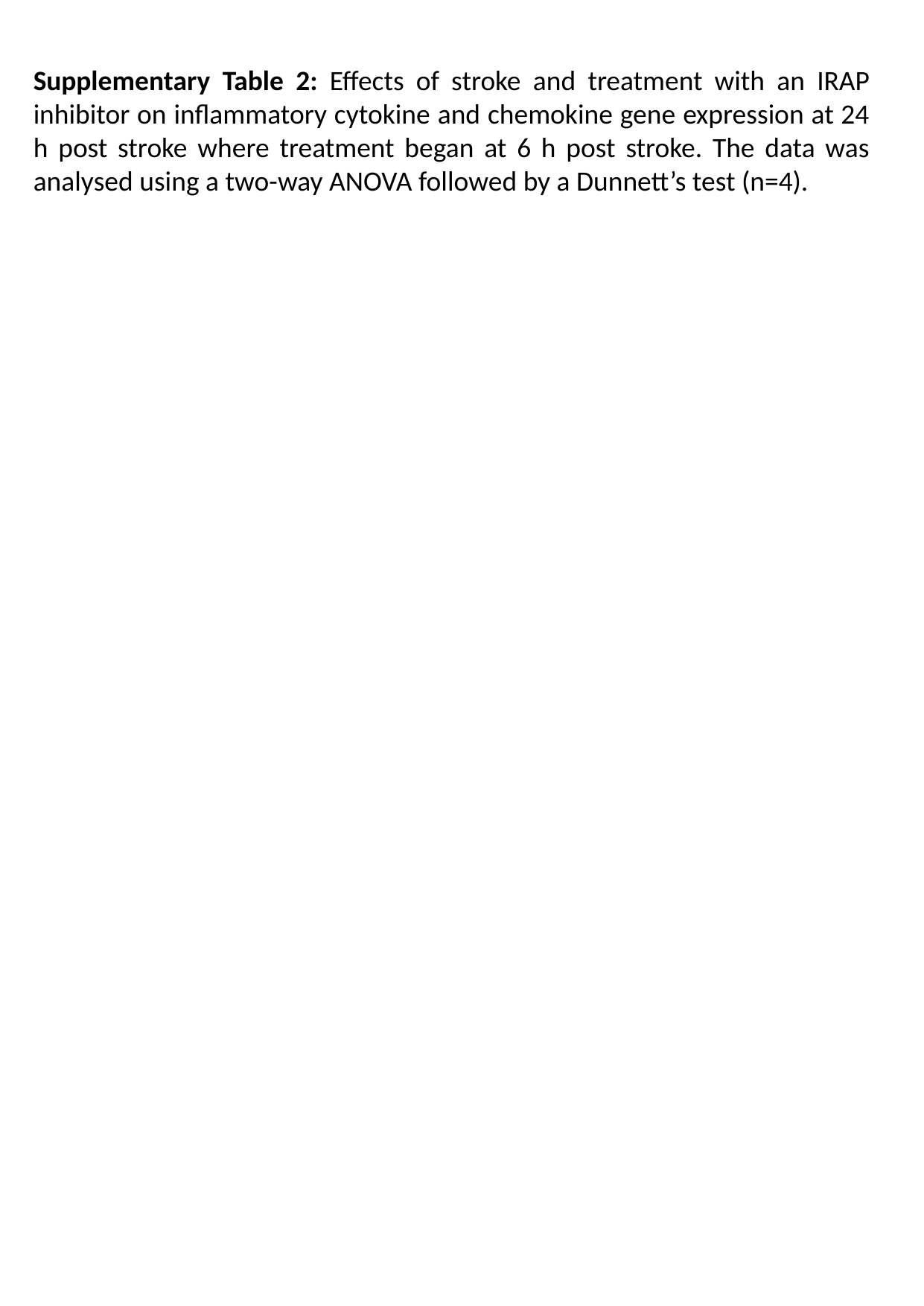

Supplementary Table 2: Effects of stroke and treatment with an IRAP inhibitor on inflammatory cytokine and chemokine gene expression at 24 h post stroke where treatment began at 6 h post stroke. The data was analysed using a two-way ANOVA followed by a Dunnett’s test (n=4).
